# Intracellular Proton Conductance of the Hepatitis C Virus p7 Protein and Its Contribution to Infectious Virus Production

**DOI:** 10.1371/journal.ppat.1001087

**Published:** 2010-09-02

**Authors:** Ann L. Wozniak, Stephen Griffin, David Rowlands, Mark Harris, MinKyung Yi, Stanley M. Lemon, Steven A. Weinman

**Affiliations:** 1 Department of Internal Medicine, University of Kansas Medical Center, Kansas City, Kansas, United States of America; 2 Department of Neuroscience and Cell Biology, Institute for Human Infections and Immunity, University of Texas-Medical Branch, Galveston, Texas, United States of America; 3 Institute of Molecular and Cellular Biology, Faculty of Biological Sciences & Astbury Centre for Structural Molecular Biology, University of Leeds, Leeds, United Kingdom; 4 Leeds Institute of Molecular Medicine, St James's University Hospital, University of Leeds, Leeds, United Kingdom; 5 Department of Microbiology and Immunology, Institute for Human Infections and Immunity, University of Texas-Medical Branch, Galveston, Texas, United States of America; 6 Center for Infectious Diseases, University of North Carolina at Chapel Hill School of Medicine, Chapel Hill, North Carolina, United States of America; The Rockefeller University, United States of America

## Abstract

The hepatitis C virus (HCV) p7 protein is critical for virus production and an attractive antiviral target. p7 is an ion channel when reconstituted in artificial lipid bilayers, but channel function has not been demonstrated *in vivo* and it is unknown whether p7 channel activity plays a critical role in virus production. To evaluate the contribution of p7 to organelle pH regulation and virus production, we incorporated a fluorescent pH sensor within native, intracellular vesicles in the presence or absence of p7 expression. p7 increased proton (H^+^) conductance in vesicles and was able to rapidly equilibrate H^+^ gradients. This conductance was blocked by the viroporin inhibitors amantadine, rimantadine and hexamethylene amiloride. Fluorescence microscopy using pH indicators in live cells showed that both HCV infection and expression of p7 from replicon RNAs reduced the number of highly acidic (pH<5) vesicles and increased lysosomal pH from 4.5 to 6.0. These effects were not present in uninfected cells, sub-genomic replicon cells not expressing p7, or cells electroporated with viral RNA containing a channel-inactive p7 point mutation. The acidification inhibitor, bafilomycin A1, partially restored virus production to cells electroporated with viral RNA containing the channel inactive mutation, yet did not in cells containing p7-deleted RNA. Expression of influenza M2 protein also complemented the p7 mutant, confirming a requirement for H^+^ channel activity in virus production. Accordingly, exposure to acid pH rendered intracellular HCV particles non-infectious, whereas the infectivity of extracellular virions was acid stable and unaffected by incubation at low pH, further demonstrating a key requirement for p7-induced loss of acidification. We conclude that p7 functions as a H^+^ permeation pathway, acting to prevent acidification in otherwise acidic intracellular compartments. This loss of acidification is required for productive HCV infection, possibly through protecting nascent virus particles during an as yet uncharacterized maturation process.

## Author Summary

The hepatitis C virus (HCV) is the most common cause of chronic liver disease. Current therapy is only partially effective and fraught with side effects. A greater understanding of viral replication and new virus particle formation is thus important for developing new therapeutic targets. The HCV p7 protein is a virally encoded protein that is absolutely required for the production of new virus particles. It behaves as an ion channel when reconstituted into artificial lipid membranes but its function in infected cells is unknown. We have examined the possibility that p7 functions as an intracellular ion channel, preventing pH gradients from developing inside the cells. We have shown that p7 serves this function and it causes a loss of acidity in multiple intracellular compartments. We demonstrate that this alkalinization is required for successful virus production. Either direct inhibition of intracellular ATPases or replacement of p7 with an alternative ion channel is able to compensate for a defect in p7 and allow active virus to be produced. Therefore, HCV uses p7 to prevent cellular acidification processes. This understanding will allow for the targeting of this mechanism with novel therapeutic agents, and offers new insights into the mechanisms of liver pathogenesis during infection.

## Introduction

Hepatitis C virus (HCV) primarily infects human hepatocytes and results in a severe liver disease manifested by chronic inflammation, progressive fibrosis and development of hepatocellular carcinoma. The virus is highly successful in evading the host innate and adaptive immune systems [1]. HCV is highly heterogeneous, leading to genotypic-dependent variations in pathogenic manifestations and responsiveness to antiviral therapy. Standard HCV therapy, consisting of interferon and ribavirin, is only partially successful. Therefore, there is great interest in the development of new classes of antiviral agents.

The HCV p7 protein is a potential antiviral target. It is not required for viral RNA replication in cell culture, yet is essential for HCV infectivity in chimpanzees [2]. It is a member of a class of viral permeability altering proteins termed “viroporins”. Viroporins are small, virally-encoded proteins that, once inserted into cellular membranes, homo-oligomerize to form pores increasing permeability to ions and small molecules [3], [4]. In many cases, this channel activity is essential for viral propagation and infectivity. Other known viroporins include human immunodeficiency virus type 1 (HIV-1) Vpu, dengue virus M protein, influenza A virus M2 protein, and poliovirus 2B [3], [4]. The p7 protein is a small *trans*-membrane protein possessing two hydrophobic membrane-spanning regions separated by a short basic loop which is conserved amongst HCV genotypes [5], [6]. HCV p7 forms a multimeric ion channel in artificial bilayers that is preferentially permeable to cations [7], [8], [9] yet has never been shown to act as an ion channel in biological membranes.

Viroporins play vital roles in cellular entry and/or exit of several viruses including the close relative of HCV, bovine viral diarrhea virus (BVDV), where its homologous p7 protein is required for infectious virus production [10]. The well characterized M2 viroporin of influenza virus plays roles both during viral entry and egress. During entry, the M2 proton channel shunts H^+^ from the acidic endosome to the virion interior, initiating uncoating of the genome and so allowing RNA replication. In certain subtypes, M2 also equilibrates the intraluminal pH of the trans-Golgi network with the cytoplasm, preventing premature conformational changes in the viral haemagglutinin (HA) during exit (reviewed in [11], [12], [13], [14]). While HCV p7 is clearly essential for efficient infectious particle formation, its exact function in the viral lifecycle is unknown. Similarly to the situation for M2, p7 has been proposed to cause a proton (H^+^) leak preventing acidification during the exocytosis of viral particles and we have previously demonstrated its ability to replace M2 in protecting HA from low pH [15]. It has thus been suggested that p7 is primarily involved in the late phase of the virus lifecycle, where it is required for the efficient release of infectious virions [16]. However, like other viroporins it is likely that p7 has multiple functions and recent evidence suggests that p7 may be involved in the earlier stages of virus production as well [17]. Moreover, mutation complementation analysis suggests that p7 interacts directly with both other viral proteins as well as host proteins essential for infectious virus production [18]. The role of p7 in the HCV lifecycle therefore remains controversial. This problem is exacerbated by the finding that amantadine, an inhibitor of the ion channel activity in p7-containing artificial lipid bilayers, is required at much higher concentrations to inhibit virus production in culture. It is further not known whether the presence of p7 is sufficient to alter pH gradients across intracellular membranes or whether p7 inhibitors efficiently block this conductance in a native system.

Here, we describe a new method allowing the H^+^ conductance of p7 to be evaluated in native, intracellular membranes and in live cells. Importantly, we specifically show that this ion channel activity is necessary for infectious virus production in culture. The results demonstrate that, when exposed to a sudden pH shift, intracellular p7-containing vesicles equilibrate pH more rapidly than vesicles lacking p7, and this activity can be blocked by p7 inhibitors in a genotype-specific manner, as seen previously [19]. The presence of p7 in Huh-7.5 cells resulted in a loss of vesicular compartment acidification, which was not observed in cells expressing the inactive p7 mutant, p7K33A/R35A (p7KR). Furthermore we show that, while viral genomes containing p7KR are unable to support infectious virus production, infectivity can be partially rescued either by preventing vesicle acidification using bafilomycin, or by complementation with influenza A M2 protein. Finally, we show that intracellular infectious virus particles display greatly increased acid sensitivity compared to extracellular virions, which were unaffected by incubation at pH 4.0; the same pH reduced intracellular infectivity by ∼100-fold. These data support the notion that p7 prevents H^+^ gradient development and that this step is a necessary part of the viral lifecycle.

## Results

### Preparation and pH Indicator Loading of p7-containing Membrane Vesicles

**Figure 1:**
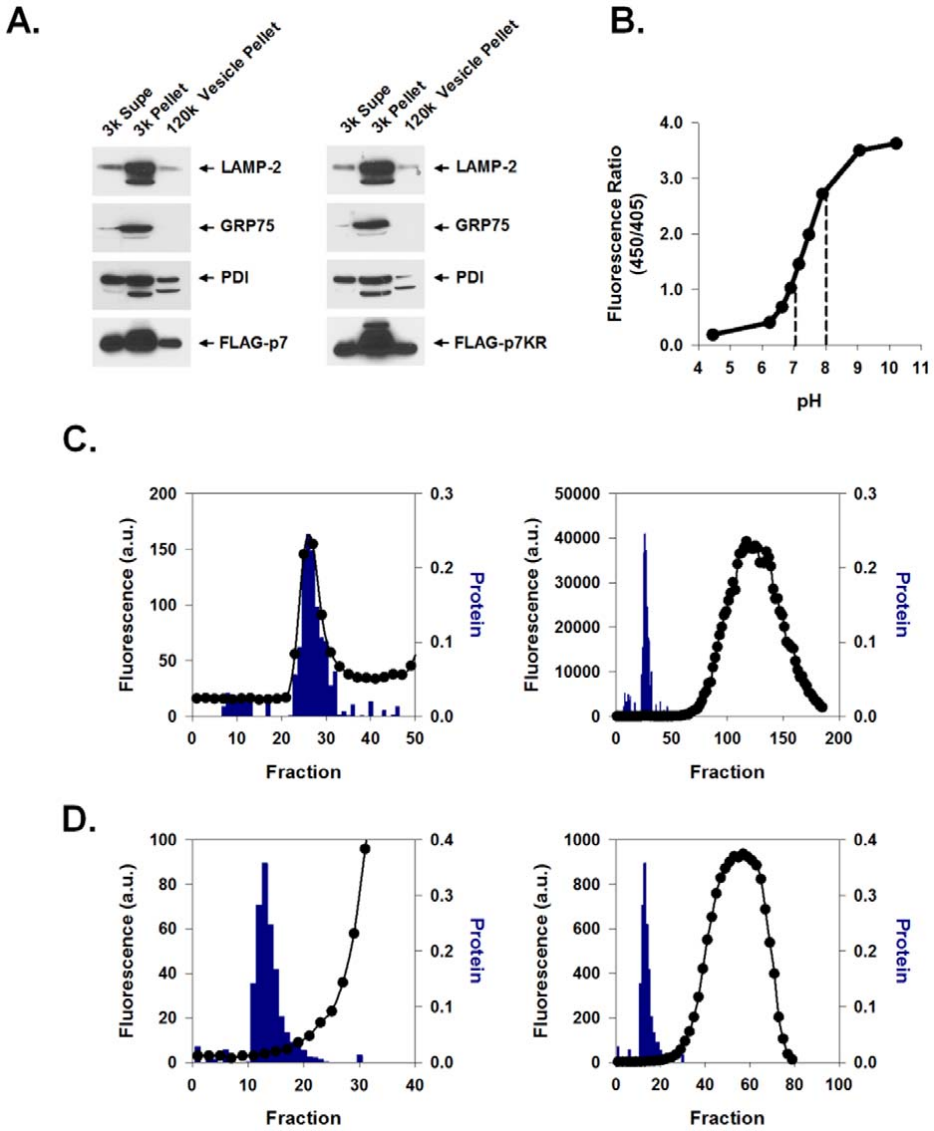
Preparation and pH indicator loading of p7-containing membrane vesicles. HEK-293FT cells were transiently transfected with either FLAG-p7 or FLAG-p7KR, homogenized 24 h later, and subjected to an initial centrifugation at 3,000× *g* followed by ultracentrifugation of the supernatant at 120,000× *g*. (**A**) Western blot of the cytosolic extract (3k Sup), heavy membrane fraction (3k Pellet) and light membrane fraction (120k pellet) showing that FLAG-p7 (left panel) and FLAG-p7KR (right panel) were present in whole cell extracts as well as in the heavy membrane and light membrane fractions. Marker proteins were LAMP-2 (lysosomal/late endosomal protein), PDI (ER chaperone) and GRP75 (mitochondrial chaperone). (**B**) HPTS (100 nM solution) was titrated to varying pH values from 4.5 to 10.5 and fluorescence emission (F) at 520 nm was determined using excitation wavelengths of 450 and 405 nm. The ratio of F_450_/_405_ is presented as a function of pH. Dotted lines represent the linear range. (**C**) Frozen 120k vesicle pellet was thawed in the presence of HPTS and re-homogenized as described in Materials and Methods. The mixture was applied to a Bio-Gel P-10 size exclusion column and eluted with constant flow. The column void volume eluted at approximately fraction 28. The right panel shows both fluorescence and protein content of fractions over the whole column profile while the left panel shows results around the void volume only with a magnified ordinate. Solid symbols (•) represent fluorescence; bars indicate protein concentration. (**D**) Protocol was similar to that shown in C except that the vesicle prep was first exposed to Triton X-100 prior to gel filtration. In this experiment the void volume eluted approximately at fraction 15. Note the absence of a fluorescence peak associated with the eluted protein.

FLAG-p7 was expressed in 293FT cells and its subcellular localization was assessed by cell fractionation. Cells were subjected to differential centrifugation as described in Materials and Methods yielding a 3,000× *g* pellet, 3,000× *g* supernatant and 120,000× *g* vesicle pellet. Confirming previous results [20], Fig. 1A, left panel, demonstrates that p7 was present in the 3,000× *g* heavy membrane pellet which also contained endoplasmic reticulum (ER), mitochondria and lysosomes as evidenced by the markers PDI, GRP75 and LAMP-2. The 120,000× *g* light membrane vesicle pellet also contained p7, lysosomes and ER and was subsequently used to measure proton (H^+^) permeability.

HCV p7 contains two hydrophobic *trans*-membrane regions separated by a short basic loop that has been suggested to be necessary for p7 ion channel activity [15], [16] and insertion into artificial lipid bilayers [21], [22]. This loop is conserved amongst HCV genotypes, further suggesting that it plays a role in p7 function. To confirm that mutation of these residues does not alter its expression, lysates from FLAG-p7KR mutant-expressing cells were analyzed. Differential centrifugation of the membrane fractions showed a similar distribution of the mutant p7 to that of wild-type p7, showing that mutation of the loop region did not alter its localization or expression (Fig. 1A, right panel).

In order to measure the proton permeability properties of p7 in native intracellular vesicles, we used the membrane impermeant pH-dependent fluorophore 8-hydroxypyrene-1,3,6-trisulfonic acid (HPTS). This compound can be used as a ratiometric pH indicator with a near-linear response between pH 7.0 and 8.0 (Fig. 1B). Isolated membrane vesicles (120,000× *g* pellet) were loaded ex vivo with HPTS and purified from free dye using a Bio-Gel P-10 size exclusion column as described in Materials and Methods. HPTS-loaded vesicles exited in the void volume and were detected as a small peak in fluorescence which corresponded to a high molecular weight protein peak (Fig. 1C, left panel, bars) that exited well before the bulk of the fluorophore eluted from the column (Fig. 1C, right panel, circles). Unloaded vesicles had no intrinsic fluorescence (data not shown). Adding Triton X-100 (final concentration of 0.5%) to the loaded vesicle homogenate prior to column purification resulted in complete HPTS release (Fig. 1D). This confirmed the intra-vesicular localization of fluorophore.

### Measurement of Vesicular pH

**Figure 2:**
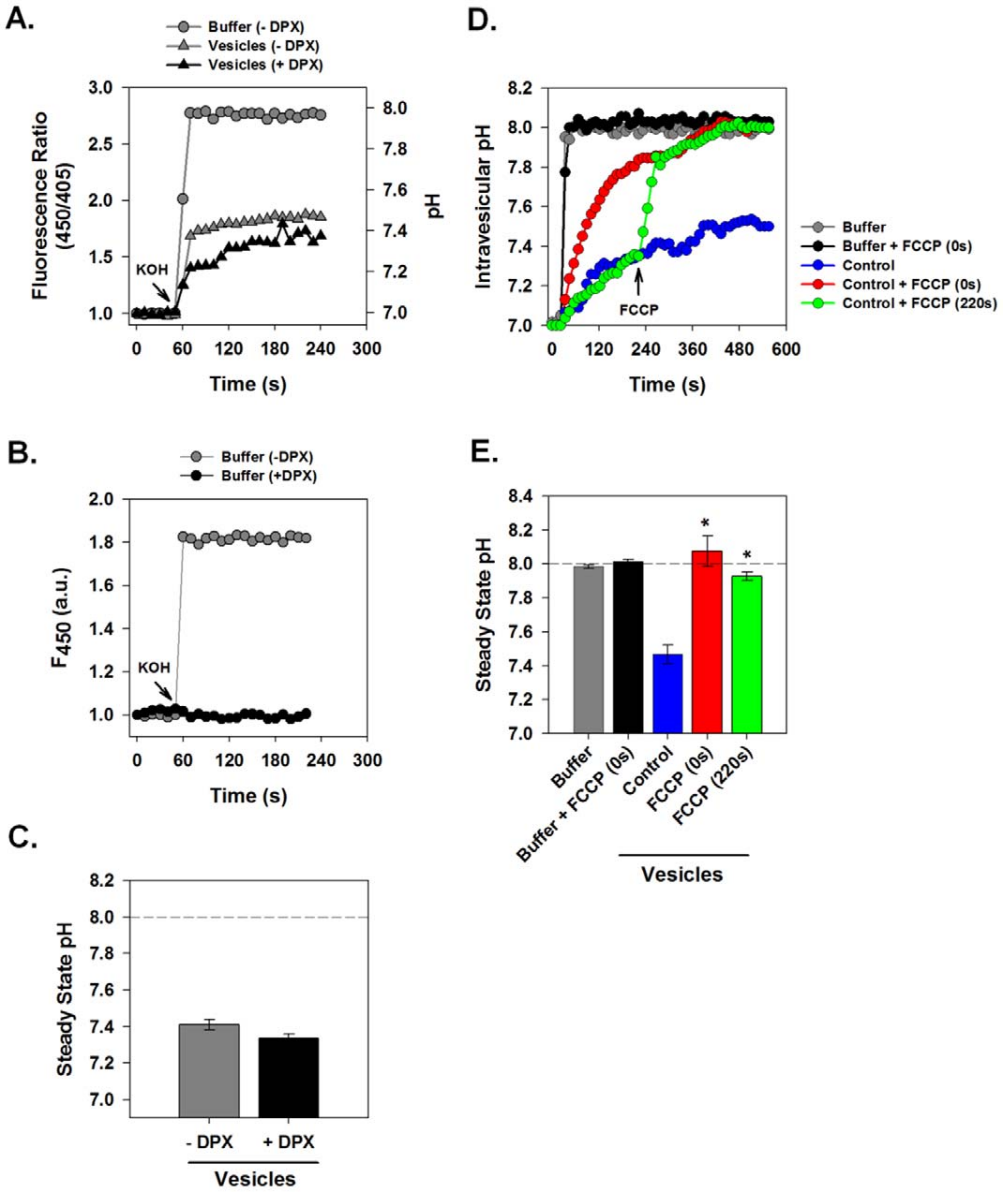
Measurement of Intravesicular pH. HPTS-loaded vesicle suspensions or HPTS solution without vesicles were subjected to rapid alkalinization by sudden injection of 10 µl of 50 mM KOH into a total volume of 100 µl. Fluorescence ratio (F_450_/F_405_) and corresponding pH was measured at 1 s intervals in a fluorescence plate reader. (**A**) Comparison of pH changes of HPTS in free solution (gray circles) vs. in suspensions of HPTS-containing vesicles (gray triangles). Free solution rapidly equilibrated to pH 8.0 while vesicles alkalinized more slowly and failed to equilibrate with the extravesicular solution pH during the observation period. Inclusion of DPX, a membrane impermeant fluorescence quenching agent (black triangles) did not affect intravesicular pH changes. (**B**) Effects of DPX on extracellular solution fluorescence. Absolute fluorescence, F_450_, was measured in HPTS solution before and after alkalinization. The presence of DPX (15 mM) completely quenched fluorescence of this fluorochrome. (**C**) Steady state pH values measured at 240 s from a series of experiments performed as in panel A. Data is presented as mean ± SE, n = 4 independent vesicle preparations. (**D**) Measurement of the effect of the protonophore FCCP on intravesicular pH changes. HPTS-loaded vesicles were incubated with or without FCCP (4 µM) added either immediately prior to (red line) or 4 min after (green line) the pH shift. FCCP had no effect on free solution pH as shown in the black and grey curves. (**E**) Steady state pH values as described for panel C. n = 4 independent membrane preparations. * indicates *P*≤0.05 compared to control vesicles.

To assess vesicular H^+^ conductance, we measured the fluorescence response of intra-vesicular HPTS to a sudden increase of extra-vesicular pH. HPTS-loaded intracellular membrane vesicles were pre-equilibrated at pH 7.0 in conductance assay buffer. Sudden addition of KOH alkalinized the extra-vesicular space, inducing a rapid pH change from 7.0 to 8.0. Fig. 2A illustrates the response of HPTS fluorescence ratio to sudden extravesicular alkalinization. When HPTS was free in solution, i.e. in the absence of vesicles, (Fig. 2A, circles), the addition of KOH rapidly alkalinized the solution from pH 7.0 to 8.0. A rapid and immediate increase in fluorescence occurred within 5 s as a pH of 8.0 was immediately reached. However, when the HPTS was present within vesicles (Fig. 2A, grey triangles), KOH addition produced an initial change in pH followed by a slower equilibration phase whereby the vesicle interior ultimately reached a steady-state pH of approximately 7.4, considerably more acidic than the extravesicular solution. This signal resulted from an intravesicular localization of HPTS because the fluorescence ratio was not affected by the addition of a membrane-impermeant fluorescence quencher, p-xylene-bis-pyridinium bromide (DPX) (Fig. 2A, black triangles). When added to the extravesicular environment, 15 mM DPX quenched extravesicular fluorescence, leaving the intravesicular signal intact. Fig. 2B shows that 15 mM DPX was sufficient to completely eliminate the pH-dependent fluorescence wavelength (F_450_) of HPTS in free solution. This confirms that the addition of DPX fully quenches extravesicular HPTS. Furthermore, rapid and full equilibration to pH 8.0 occurred after disruption of vesicles by Triton X-100 (data not shown).

We postulated that vesicles prepared from normal 293FT cells might have delayed pH equilibration with the external solution because H^+^ conductance was rate limiting in the face of ionic transport processes that tended to produce a net acidification. To test this hypothesis we examined the effect of a proton ionophore on pH equilibration in this system. The addition of FCCP (carbonyl cyanide 4-(trifluoromethoxy) phenylhydrazone) immediately prior to vesicle alkalinization resulted in a more rapid intravesicular pH rise that reached full equilibration with the extravesicular solution (Fig. 2D–E, red). Furthermore, addition of FCCP during the period of pH equilibration caused a rapid alkalinization to full pH equilibrium (Fig. 2D–E, green). As expected, FCCP had no effect on HPTS fluorescence in bulk solution. This demonstrates that H^+^ efflux is limited by the intrinsic H^+^ permeability/conductance of the membrane in these vesicles.

### Conductive Properties of p7 Proteins from Different HCV Strains Expressed in Native Intracellular Membranes

**Figure 3:**
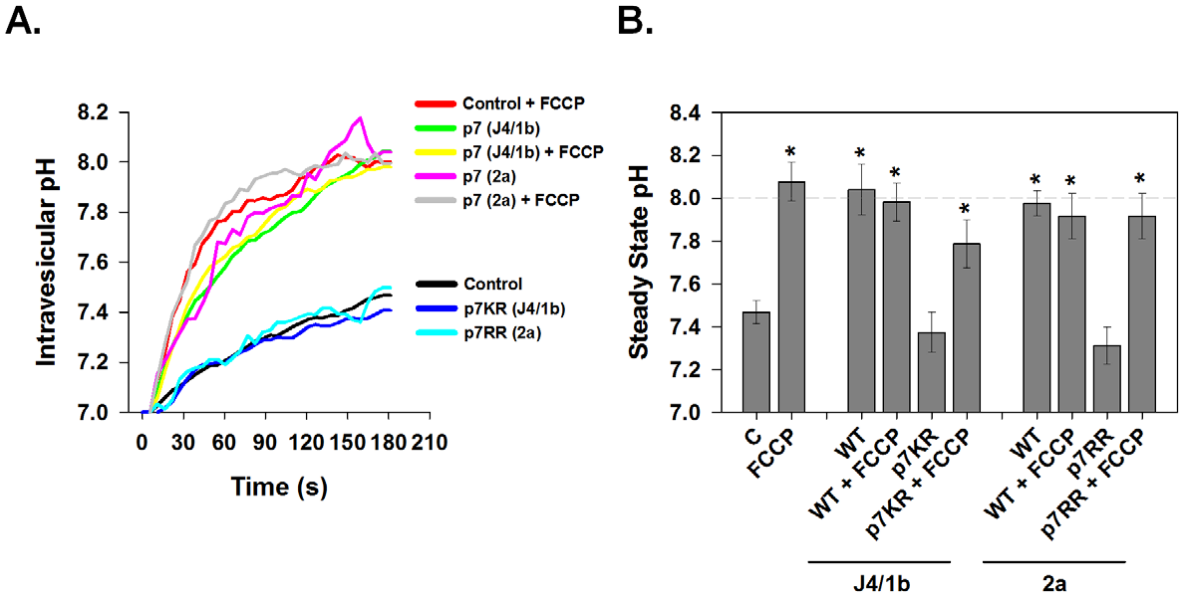
Conductive properties of HCV p7 proteins. Vesicle pellets were isolated from HEK-293FT cells that were transfected with either genotype 1b p7 (J4 sequence), genotype 2a p7 (JFH1 sequence), the corresponding channel inactive mutants p7K33A/R35A (p7KR) and p7R33A/R35A (p7RR) or mock control vector. Vesicles were loaded with HPTS as described in Materials and Methods and subjected to rapid alkalinization by sudden injection of KOH at time zero according to the protocol shown in Fig. 2. Where indicated, FCCP (4 µM) was added immediately prior to the addition of KOH. (**A**) A single representative experiment is shown demonstrating the time course of intravesicular pH changes in control, p7- or mutant p7-expressing vesicles. (**B**) The average steady state pH change at 200 s was determined as in A, in response to extravesicular alkalinization. Data is presented as mean ± SE. n  =  a minimum of 6 independent vesicle preparations for each condition. * indicates *P*≤0.05 compared to control vesicles with no FCCP.

p7 sequences have been suggested to promote virus production in a genotypic-specific manner [16], however it is unknown if this is related to ion channel function. We therefore examined the ability of p7 proteins from different HCV genotypes to alter membrane H^+^ permeability. Native intracellular membrane vesicles (120,000× *g* pellet) expressing either genotype 1b (J4 isolate, “FLAG-p7”) or 2a (JFH-1 isolate, “FLAG-JFH1p7”) p7 displayed a significant increase in H^+^ permeability when compared to vesicles from control cells (Fig. 3) and stabilized at an intra-vesicular pH of approximately 8.0. The addition of FCCP had no additional effect on the efflux of H^+^ from p7-containing vesicles showing that conductive H^+^ flux was no longer rate limiting for pH equilibration.

The membrane spanning domains of p7 are separated by a basic loop which is conserved amongst HCV genotypes further suggesting it plays a role in p7 function. We have previously shown that mutating these basic residues to alanine (FLAG-p7K33A/R35A mutant and FLAG-JFH1p7R33A/R35A mutant) renders p7 incapable of protecting HA from acidic pH [15], or serving as a permeation pathway in *in vitro* liposome-based assays for channel function [22]. Yet its direct effect on p7-induced H^+^ permeability is unknown. Vesicles isolated from cells expressing the mutated FLAG-p7KR or FLAG-JFH1p7RR displayed a H^+^ conductance, similar to that of control vesicles from untransfected 293FT cells. Furthermore, the addition of FCCP was still able to restore rapid and full pH equilibration demonstrating that the failure of the mutant p7 to cause rapid pH equilibration was due to a lack of an effect on proton conductance. This result indicates that these conserved amino acids are in fact necessary for p7-induced H^+^ conductivity.

### HCV p7-Induced H^+^ Conductance is Sensitive to Known Viroporin Inhibitors

**Figure 4:**
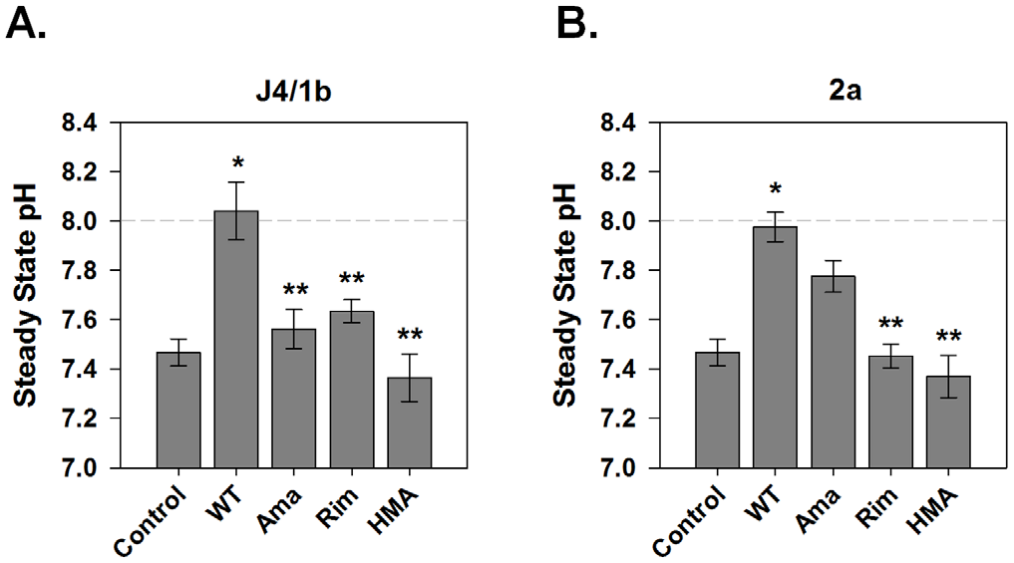
Sensitivity of p7-associated pH changes to known viroporin inhibitors. HPTS loaded vesicles were subjected to rapid alkalinization by sudden injection of KOH as described for Figs. 2 and 3. The H^+^ conductance induced by the J4/1b p7 (**A**) and JFH1/2a/p7 (**B**) was inhibited by several viroporin inhibitors. Data is presented for each of the two genotypes as mean ± SE of the steady state pH change observed in control, p7-expressing vesicles (WT) and in p7 containing vesicles in which inhibitor compounds (1 µM) were added immediately prior to the pH shift. Ama  =  amantadine, Rim  =  rimantadine and HMA  =  hexamethylene amiloride. n  =  a minimum of 3 independent vesicle preparations. * indicates *P*≤0.05 compared to control, ** indicates *P*≤0.05 compared to wild-type.

Several viroporin inhibitors have been shown to abolish p7 ion channel activity in artificial lipid bilayers [7]–[9], as well as to inhibit infectious particle production in a genotype-dependent manner [19]. However, it is not known whether they function to block intracellular H^+^ conductance. To confirm the specificity of our observation that p7 induces a significant H^+^ conductance, we assessed the effect of several candidate p7 inhibitors. Vesicles incubated with 1 µM amantadine, a concentration shown to inhibit p7 in artificial bilayers [7], [22], [23] as well as specifically inhibit the M2 viroporin of influenza [13], [24], reduced the H^+^ conductance induced by the J4/1b p7 to that of control vesicles (Fig. 4A). Inhibition of J4/1b p7 H^+^ conductance was also seen with the amantadine derivative, rimantadine, as well as hexamethylene amiloride (HMA). In agreement with our previous observations *in vitro* and in the context of infectious virus [19], inhibitor sensitivity varied according to p7 sequence; H^+^ conductance induced by the JFH1/2a p7 was eliminated by rimantadine and HMA, yet was insensitive to amantadine (Fig. 4B). The addition of FCCP induced an H^+^ conductance despite the presence of these inhibitors, indicating that the compounds did not alter vesicle stability or have a direct effect on HPTS fluorescence (data not shown).

### Effect of p7 on Intracellular pH in HCV Replicon-bearing Cells

**Figure 5:**
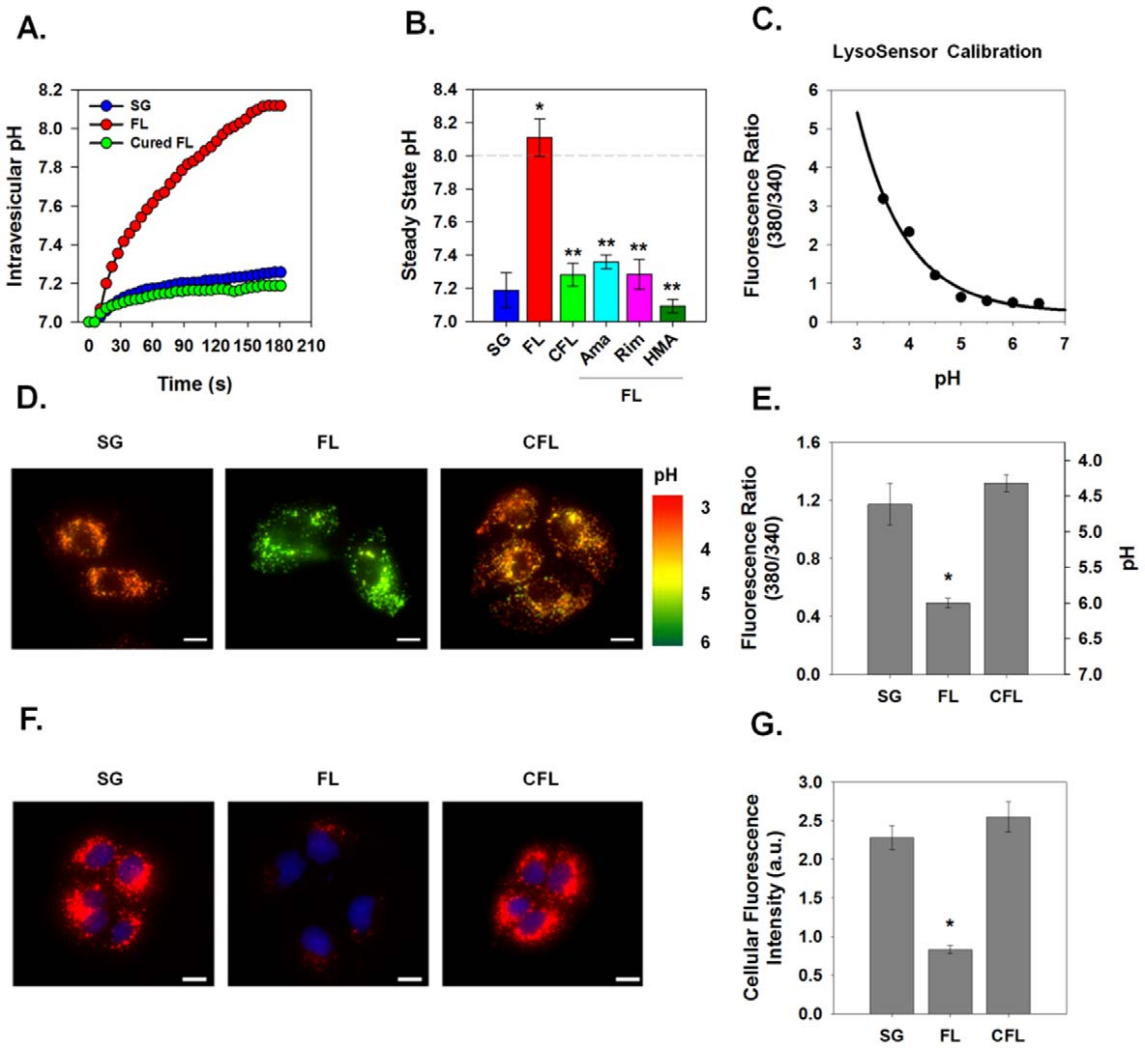
Effect of p7 on intracellular pH in HCV replicon-bearing cells. Intravesicular pH was measured in vesicles isolated from HCV replicon bearing cells that were subjected to rapid alkalinization as shown for Fig. 2. (**A**) A single representative experiment is shown demonstrating the time course of intravesicular pH changes in vesicles isolated from full-length (FL), sub-genomic (SG) and cured full-length (CFL) HCV replicons. (**B**) The average steady state pH change at 180 s in response to extravesicular alkalinization was determined. Data is presented as mean ± SE. n  =  a minimum of 6 independent vesicle preparations for each condition. Inhibitor compounds were added immediately prior to the pH shift as in Fig. 4.* indicates *P*≤0.05 compared to control, ** indicates *P*≤0.05 compared to full-length. (**C**) Free solution calibration of LysoSensor Yellow/Blue DND-160. Fluorochrome (100 nM) was dissolved in HEPES solution and titrated to various pH values. Aliquots (750 µl) were placed in a 25 mm cover slip chamber and the fluorescence was measured using a Nikon TiE fluorescence microscope at excitation 380 emission 525 (wavelength 1) and excitation 340 emission 440 (wavelength 2). Ratio is presented as wavelength 1/wavelength 2. (**D**) Sub-genomic (SG), full-length (FL) and cured full-length (CFL) HCV replicon bearing cells were loaded with the ratiometric pH sensor, LysoSensor Yellow/Blue DND-160, and imaged at the 2 wavelength pairs used for the calibration in panel C. Fluorescence ratio images are presented where the fluorescence ratio at each pixel was mapped to a pseudocolor representation based on the calibration curve. (**E**) Mean vesicular pH as determined from the fluorescence ratio images in multiple experiments as in panel D is shown. Data is presented as mean ± SE of cytosolic fluorescence ratio with the corresponding pH scale indicated on the right. Data represents the average of 25 cells in each of 4 independent cell preparations with the mean of each individual cell preparation counting as n = 1.* indicates *P*≤0.05 compared to sub-genomic replicons. Scale bar represents 20 µm. (**F**) Presence of highly acidic organelles was assessed in HCV replicon-bearing cells by incubation for 30 min with LysoTracker Red DND-99 (100 nM). Nuclei were counterstained with DAPI. Excitation and emission wavelengths for LysoTracker Red were excitation 560 nm and emission 607 nm, represented as red, and excitation 380 nm and emission 440 nm for DAPI, represented as blue. Acidic organelles appear as red structures. Scale bar represents 20 µm. (**G**) Total LysoTracker cellular fluorescence for experiments conducted as in panel F is shown. Data are presented as mean ± SE of total cellular LysoTracker fluorescence. Data represents the average of at least 25 cells in a minimum of 3 independent cell preparations with the mean of each individual cell preparation counted as n = 1. * indicates *P*≤0.05 compared to sub-genomic replicons.

To overcome limitations presented by a transfection-based system, an HCV replicon system was utilized. We studied Huh7-derived cell lines harboring RNAs from a third HCV genotype 1a strain (H77) [25], [26]. Replicon cell lines contained stably replicating sub-genomic RNA (NS2 – NS5b) or full-length RNA (Core – NS5b). A cured full-length replicon cell line, in which the HCV RNA had been eliminated by prior treatment with interferon, served as a control. The response to a *trans*-vesicular pH gradient was greatly enhanced in vesicles prepared from cells containing the full-length replicon, which rapidly equilibrated to pH 8.0, when compared to the cells containing the sub-genomic replicon that does not express p7 (Fig. 5, A–B). Vesicles isolated from the cured replicon cells displayed a similar conductance pattern as the vesicles isolated from sub-genomic replicon cells. Isolated full-length replicon cell vesicles also showed specific viroporin-mediated H^+^ permeability; as seen previously [19], the H77 p7-induced H^+^ conductance was inhibited by amantadine, rimantadine and HMA. Pretreatment of vesicles from full-length replicon cells with 1 µM inhibitor resulted in almost 100% inhibition of H^+^ conductance.

p7 thus functions as proton permeation pathway in vesicles prepared from native, intracellular membranes and has inhibitor sensitivities identical to those determined in bilayers and artificial liposomes [19]. To determine if this activity is present in live cells, we incubated replicon cells with LysoSensor Yellow/Blue DND-160, a pH probe that exhibits a pH-dependent shift in fluorescence upon acidification. As shown in the calibration curve in Fig. 5C, LysoSensor Yellow/Blue DND-160 can be efficiently used to monitor intracellular vesicular pH of live cells. Live cell ratiometric imaging of HCV replicon-bearing cells showed punctuate vesicles of varying size (Fig. 5D). Cells containing full-length HCV replicon RNAs, expressing p7, had a net alkalinization of these vesicular structures and an average vesicular pH of nearly 6.0, whereas both sub-genomic and cured cells possessed an intra-vesicular pH of approximately 4.5 (Fig. 5D–E).

To confirm these results with an alternate pH probe, we used LysoTracker Red DND-99, a fixable fluorescent probe that has high selectivity for acidic organelles. Once inside acidic compartments, this fluorophore will fluoresce red while more neutral compartments will remain non-fluorescent. We found that cells containing the replicating full-length HCV RNA demonstrated a significant loss in acidic organelle staining with punctuate red fluorescence (Fig. 5F–G) compared to both sub-genomic and cured HCV replicon cells. These data strongly support the notion that p7 acts as a pH equilibrating H^+^ channel *in vivo*.

### p7 Induces Channel Activity within Infected Cells

**Figure 6:**
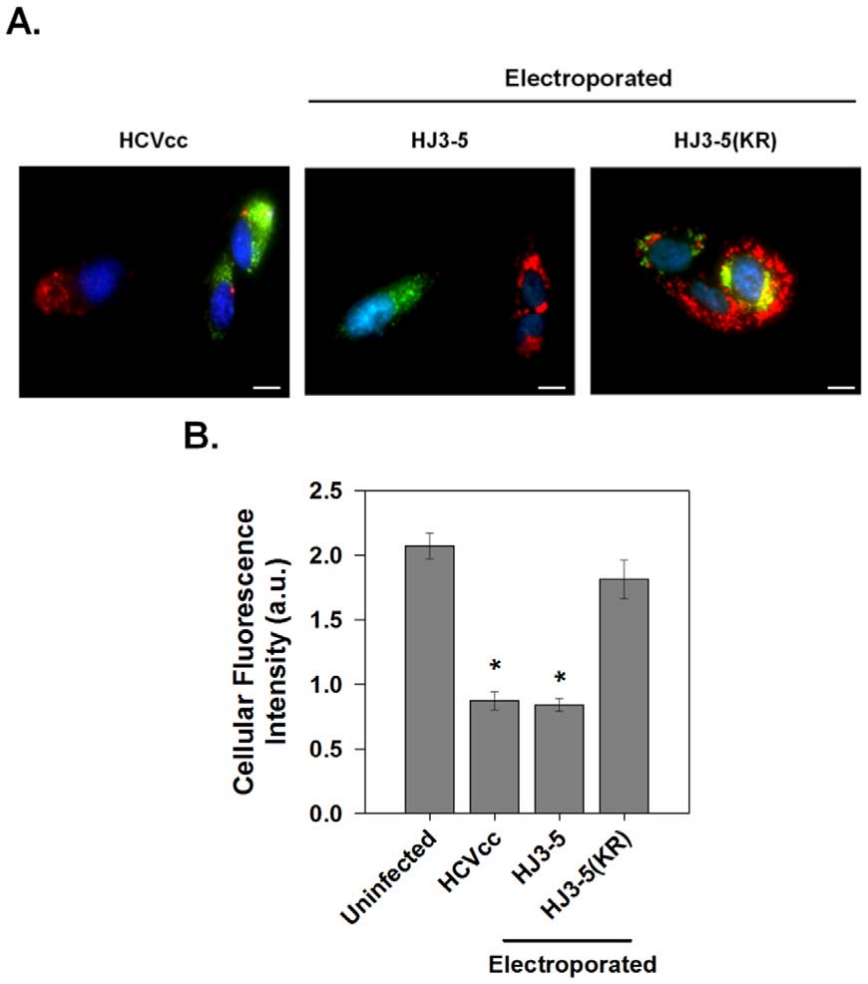
p7 channel activity within infected cells. (**A**) Huh-7.5 cells were infected with HJ3-5 virus (HCVcc) or electroporated with parental HJ3-5 or mutant HJ3-5(KR) HCV RNA and assayed after 3 days. Cells were subsequently incubated with LysoTracker Red DND-99 as described in Materials and Methods and viral replication was detected by immunostaining for core protein (green). Representative images showing both infected and non-infected cells are shown for each condition. Scale bar represents 20 µm. (**B**) Mean ± SE of total cellular LysoTracker fluorescence. Total magnitude of red fluorescence was calculated separately for non-infected cells and for cells infected with each of the different virus constructs as described for Fig. 5G. Data represents the average of at least 6 cells in each of 4 independent cell preparations with the mean of each individual cell preparation counted as n = 1. * indicates *P*≤0.05 compared to uninfected cells.

Given that p7 acts as a H^+^ channel in cells containing HCV replicons, we sought to determine whether this conductance was also present during HCV infection. Huh-7.5 cells were infected with the parental HJ3-5 virus at an MOI of 0.1, and stained with LysoTracker Red DND-99 to label acidic compartments. Infected cells were identified by immunofluorescence staining for core protein. Infection of Huh-7.5 cells with HJ3-5 resulted in a global loss of highly acidic organelles and compartments (Fig. 6A-B, HCVcc). To ensure that the loss of acidic structures seen during HJ3-5 infection was due to p7 activity, we tested the effect on pH equilibration following electroporation of cells with either HJ3-5 RNA containing the wild-type or mutant p7KR sequence (Fig. 6). Unlike the parental HJ3-5 viral RNA, the p7KR mutant was unable to alter intracellular pH, confirming that p7 acts as a proton channel during HCV infection.

### Effects of Viroporin Inhibitors on Vesicular pH and Infectious Virus Production

**Figure 7:**
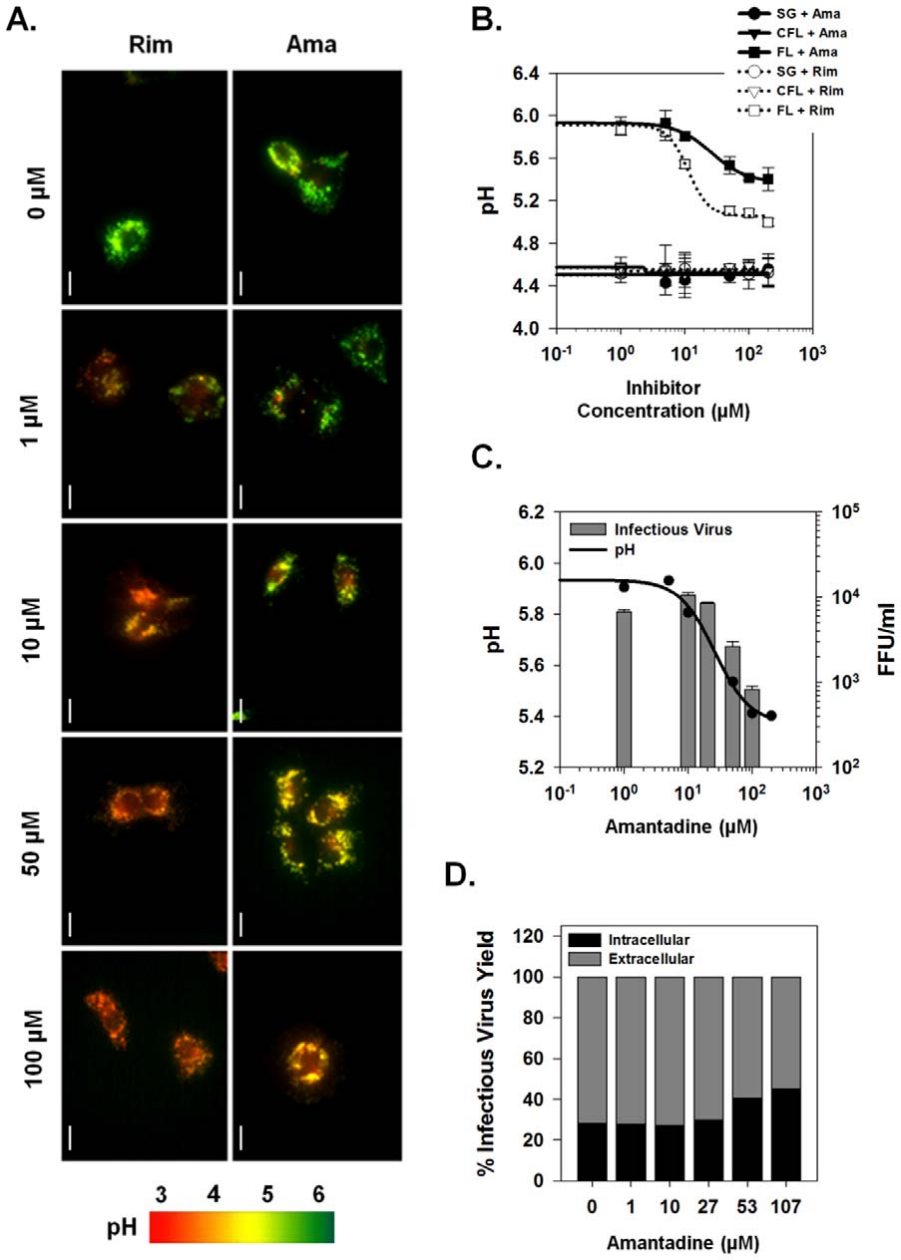
Effects of viroporin inhibitors on intracellular vesicular pH and infectious virus production. (**A**) The ability of inhibitors to affect p7 channel function *in vivo* was assessed in full-length replicon bearing cells loaded with LysoSensor Yellow/Blue DND-160. Amantadine or rimantadine were added directly to LysoSensor loaded cells and the cells were imaged after 5 min. Fluorescence ratio images are presented as for Fig. 5D and pH is represented by pseudocolor mapping as indicated. Scale bar represents 20 µm. (**B**) Average pH changes from a minimum of 3 experiments performed as in panel A. pH values were determined from fluorescence ratios by interpolation on the calibration curve as shown in Fig. 5. FL  =  full-length, SG  =  sub-genomic and CFL  =  cured full-length HCV replicons. (**C**) Comparison of the effect of various concentrations of amantadine on both intravesicular pH (data from panel B) and infectious virus present in the extracellular culture medium. For measurement of virus titers, Huh-7.5 cells were incubated with the indicated concentration of amantadine for 24 hours prior to collection of medium and tittering by the FFU assay as described in Materials and Methods. (**D**) Intra- and extracellular infectious virus production after amantadine treatment. Huh-7.5 cells were infected with HJ3-5 virus and treated with amantadine as described in Materials and Methods. Medium was collected for determination of infectious virus titer by FFU assay (Extracellular), and cell pellets were washed, disrupted by freeze-thaw and then assayed similarly (Intracellular). Bars represent the proportion of the total infectious virus yield that was present in each compartment.

We have shown that the channel activity of J4/1b, JFH-1/2a and H77/1a p7 proteins, when present in isolated membrane vesicles, is blocked by several viroporin inhibitors including amantadine and rimantadine. However, p7 channel inhibitors have yet to demonstrate clinical efficacy in the treatment of chronic hepatitis C, suggesting the possibility that the channel present in intact cells may not be as sensitive to these inhibitors. We therefore examined whether the p7 effects on vesicular pH in live cells could be reversed by channel inhibitors. Full-length replicon cells were loaded with LysoSensor Yellow/Blue DND-160 and treated with either amantadine or rimantadine. As shown previously, basal vesicular pH of full-length replicon cells was nearly 6.0, markedly more alkaline than sub-genomic or cured full-length replicon-bearing cells (Fig. 7A–B). Treatment of full-length replicons with increasing concentrations of both amantadine and rimantadine was able to partially restore vesicular acidic pH within 5 min of treatment while sub-genomic replicons and cured cells were unaffected by inhibitor treatment. Consistent with our previous observations [19], rimantadine was a more potent inhibitor (EC_50_≈15 µM) than amantadine (EC_50_≈75 µM), but in both cases, the concentration required to alter channel function in live cells was considerably higher than for isolated membrane vesicles, and full restoration of acidic compartments was not achieved. Rimantadine was able to re-acidify vesicular compartments to approximately pH 5.0 and amantadine to roughly 5.5 (Fig. 7B).

To explore the involvement of p7 H^+^ channel activity in virus production, we examined whether a direct correlate existed between the re-acidification achieved by blocking p7 channel activity with specific inhibitors and the observed reduction in infectious virus production and/or release. Huh-7.5 cells were infected with HJ3-5 virus and cultured in medium supplemented with varying concentrations of amantadine as described in Materials and Methods. Fig. 7C–D shows the titer of infectious virus released into the extracellular supernatant as well as the ratio between intracellular and extracellular virus after amantadine treatment. Consistent with replicon LysoSensor studies, amantadine at 1 µM, a concentration that nearly completely blocked p7-induced H^+^ conductance in isolated vesicles, had no effect on the release of infectious virus (Fig. 7C, bars), Yet, similar to previous results with both amantadine and rimantadine [19], increasing concentrations of amantadine reduced the release of infectious virus. Interestingly, the ability of amantadine to block virus production correlated directly with its ability to reverse the p7-induced loss of acidic intra-organelle pH (Fig. 7C, compare line and bars). As previously reported, treatment with 100 µM amantadine for 24 h did not result in any cellular toxicity [19]. This result confirms the existence of a relationship between the antiviral effect of amantadine and its ability to specifically inhibit intracellular p7 channel activity.

### p7 Channel Activity is Required for Production of Infectious Virus

**Figure 8:**
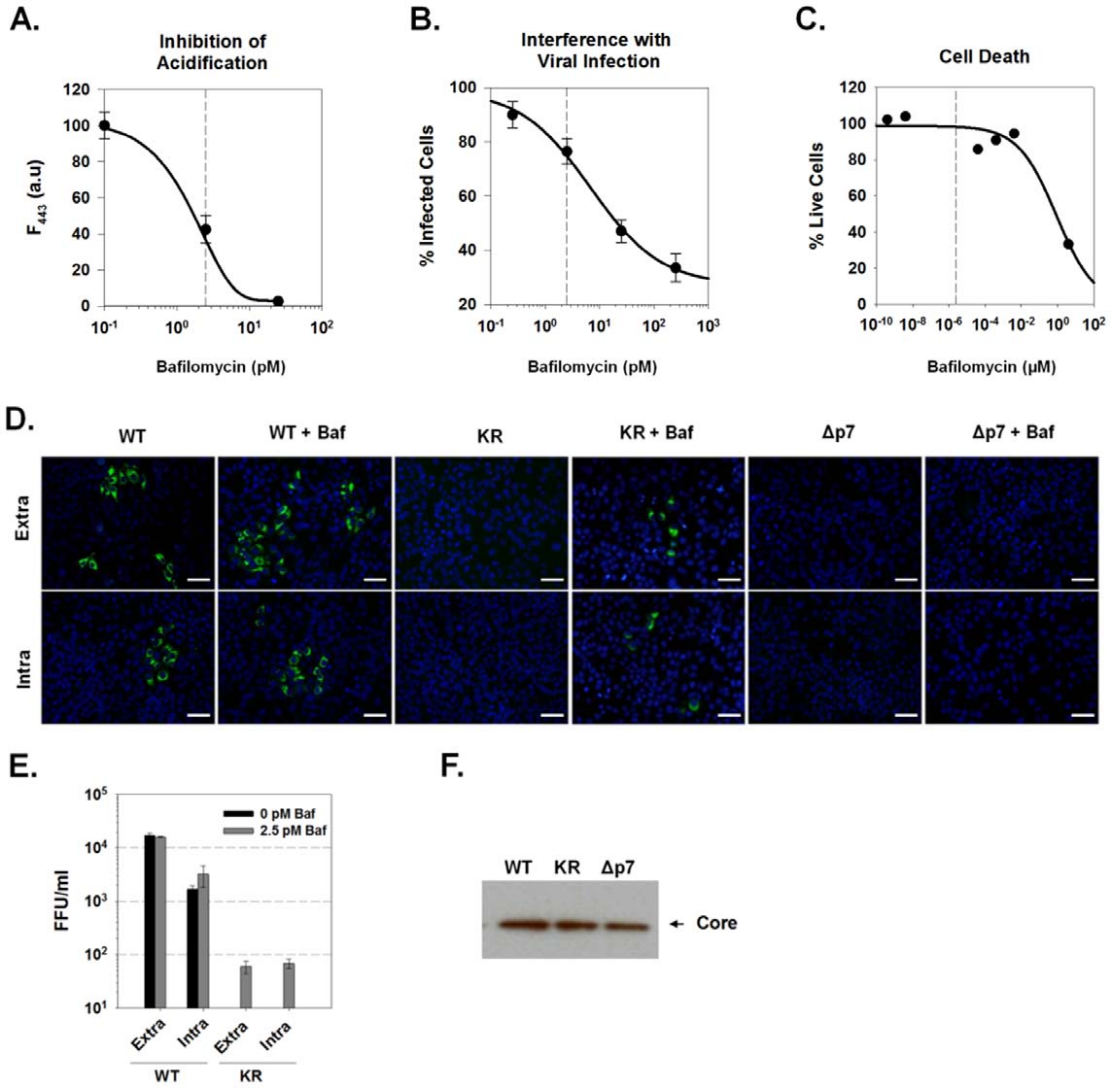
Bafilomycin A1 rescue of infectious virus production. (**A**) Concentration dependence of bafilomycin A1 on intravesicular pH in Huh-7.5 cells. Cells were loaded with the single wavelength pH indicator, LysoSensor Green DND-189, and treated with various concentrations of bafilomycin A1 as indicated. The increase in vesicular pH was measured as a decrease in fluorescence intensity at Ex/Em 443/510. Each data point represents mean ±SE from a minimum of 10 cells. (**B**) HCVcc (MOI 1.0) was used to infect naïve Huh-7.5 cells in the presence of varying concentrations of bafilomycin A1. Cells were then fixed and immunostained for core protein 3 days later. Relative infectivity was determined by measuring the intensity of core immunostaining normalized to cell number (see methods). (**C**) Cytotoxicity of bafilomycin A1 in Huh7.5 cells. Cells were cultured in the presence of various concentrations of bafilomycin A1 for 72 hrs. Cell viability was determined using CellTiter-Blue Reagent (see methods). Data are presented as the number of live cells, with untreated (no bafilomycin A1) set to 100%. (**D**) Huh-7.5 cells were electroporated with parental HJ3-5, mutant HJ3-5(KR) or HJ3-5(Δp7) HCV RNA and allowed to grow for 96 h. The cells were then treated with 2.5 pM bafilomycin A1 for 24 h after which time, intra and extracellular virus was collected and used to infect naïve Huh-7.5 cells as described in Materials and Methods. Wild-type virus supernatants were diluted 10-fold, KR and Δp7 supernatants were undiluted. These cells were immunostained for core protein (green). Example images of the formation of FFUs in each condition are presented. The scale bar represents 200 µm. (**E**) Titers of intra- and extracellular virus production in the presence or absence of 2.5 pM bafilomycin were determined from 4 experiments conducted as in panel D. No FFU were detected in the KR virus in the absence of bafilomycin in any of the experiments. (**F**) Western blot for the presence of core protein in whole cell lysates prepared from Huh-7.5 cells electroporated with WT (HJ3-5), KR (HJ3-5(KR)) or Δp7 (HJ3-5(Δp7)) HCV RNA.

To definitively determine whether the p7-induced loss of vesicle acidification contributes to infectious virion production, we determined whether preventing this acidification pharmacologically using the vATPase inhibitor, bafilomycin A1, could compensate for the p7KR defect. To perform this experiment it was necessary to first confirm that vesicular acidification could be inhibited without cellular toxicity or interference with the ability to assay for infectious virus. We determined the concentration dependence at which bafilomycin A1 affects vesicular acidification, virus entry and cell death as described in Materials and Methods (Fig. 8A–C). At 2.5 pM, bafilomycin A1 was not cytotoxic, did not significantly inhibit viral entry, yet significantly alkalinized vesicular acidic compartments to a similar extent as seen during HJ3-5 infection where an alternative pH sensitive fluorochrome of similar pKa value was used.

Huh-7.5 cells were then electroporated with the parental HJ3-5 virus RNA, the channel-inactive p7 mutant, HJ3-5(KR), or an RNA in which the p7 sequence had been removed by an in-frame deletion, HJ3-5(Δp7). After four days, the media was replaced with fresh media containing 2.5 pM bafilomycin A1. Twenty-four hours later, the cells were harvested and the titer of both intra- and extracellular infectious virus was determined by inoculation onto naïve Huh-7.5 cells. Bafilomycin A1 treatment of cells electroporated with the parental HJ3-5 virus RNA had no effect on the production of infectious virus, as intra- and extracellular virus titers remained ∼10^3^ and ∼10^4^ FFU/ml, respectively (Fig. 8D–E). As expected, cells electroporated with either the mutant HJ3-5(KR) or HJ3-5(Δp7) RNAs showed no evidence of either extracellular or intracellular infectious virus production. However, treatment with 2.5 pM bafilomycin A1 was able to partially rescue infectious virus production by the KR mutant, resulting in approximately 10^2^ FFU/ml in both the intracellular and extracellular compartments. Replication foci were noticeably smaller in cells inoculated with the rescued HJ3-5(KR) virus compared to the parental virus, consistent with a defect in cell-to-cell spread of virus within the naïve cells in the absence of drug treatment. Antibody to CD81 prevented infection by this HJ3-5(KR) virus, suggesting that it enters cells via the well characterized HCV receptor mechanism (data not shown). These results thus demonstrate that p7 H^+^ channel activity is essential for the production of infectious HCV, both within the cell and in cell culture supernatant fluids. Importantly, infectious virus production could not be rescued from HJ3-5(Δp7)-electroporated cells by bafilomycin A1 treatment, suggesting that other, non-H^+^ channel p7 functions are likely to be required for the assembly and release of infectious virus. Equal expression of HCV core protein was detected for each of these viral constructs, indicating that each of these RNAs is capable of replicating following electroporation into cells (Fig. 8F).

### Influenza M2 protein Can *Trans*-complement an HCV p7 Channel Mutant

**Figure 9:**
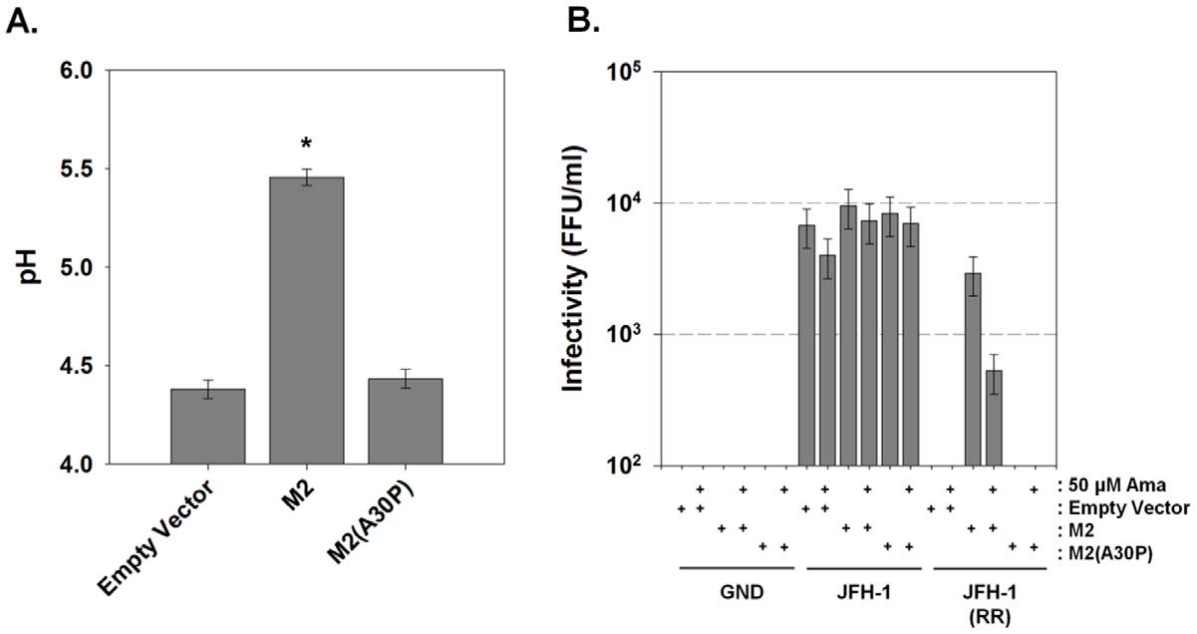
Influenza M2 protein can trans-complement an HCV p7 channel mutant. (**A**) Huh-7.5 cells were loaded with the ratiometric pH sensor LysoSensor Yellow/Blue DND-160 and imaged as described in Materials and Methods. Mean vesicular pH as determined from the fluorescence ratio in multiple experiments is shown. Data is presented as pH ± SE. Data represents the average of 20 cells in each of 3 independent cell preparations with the mean of each individual cell preparation counting as n = 1. * indicates *P*≤0.05 compared to empty vector. (**B**) Huh-7.5 cells were co-electroporated with mCherry-M2 or the channel inactive mCherry-M2(A30P) and one of three HCV RNAs: wild-type JFH-1, channel inactive JFH-1(RR) or polymerase null JFH-1(GND) HCV RNA as described in Materials and Methods. Supernatants were collected and assayed for infectious virus titer by FFU determination. Where indicated, cells were treated with 50 µM amantadine for the final 24 hrs prior to harvesting the medium. Data are represented as mean ± SE from three independent experiments.

As bafilomycin A1 partially rescued infectious virus production for the channel-inactive p7KR mutant, we investigated whether the influenza A M2 (IAV M2) proton channel might act similarly. We first examined the ability of M2 to alter pH gradients in live cells. Huh-7.5 cells were transfected with M2 or the channel inactive M2, M2(A30P) [27] and loaded with LysoSensor Yellow/Blue DND-160. The presence of M2 caused a net alkalinization of highly acidic vesicular structures measuring an average pH of 5.5 while cells expressing an empty vector or the channel inactive M2(A30P) measured an intravesicular pH of approximately 4.3 (Fig. 9A).

To assess whether M2 H^+^ channel activity was specifically able to rescue a p7-deficient HCV, we exploited the genotype-dependent sensitivity of p7 to amantadine. Thus, we assessed the ability of an amantadine-sensitive M2 protein to rescue the amantadine resistant genotype 2a JFH-1 HCV, thereby utilizing amantadine sensitivity to identify an M2-specific effect. Huh-7.5 cells were co-electroporated with plasmids encoding the amantadine-sensitive IAV M2 protein or the channel inactive M2, M2(A30P), along with wild-type JFH-1, JFH-1(RR) or polymerase mutant, JFH-1(GND) RNA. Following amantadine treatment, yields of extracellular infectious virus were assessed. Cells electroporated with JFH-1 RNA produced approximately 10^4^ FFU/ml and the release of infectious virus from JFH-1-electroporated cells was not affected by the expression of M2, M2(A30P) or the presence of 50 µM amantadine (Fig. 9B). JFH-1(RR) RNA produced no detectable infectious virus, however, co-electroporation of JFH-1(RR) and IAV M2 resulted in a partial rescue of infectious virus production, yielding between 10^3^ and 10^4^ FFU/ml. Unlike JFH-1 infected cells, however, the release of infectious virus by the M2 *trans*-complemented JFH-1(RR) mutant was partially inhibited by amantadine. In addition, the M2 mutant, M2(A30P) was unable to rescue the JFH-1(RR) mutation. This result confirms the requirement for H^+^ channel activity for virus production from the HJ3-5 chimeric virus in which the p7 sequence is of genotype 1a origin.

### Exposure to Acidic pH Renders Intracellular Infectious Virus Non-infectious

**Figure 10:**
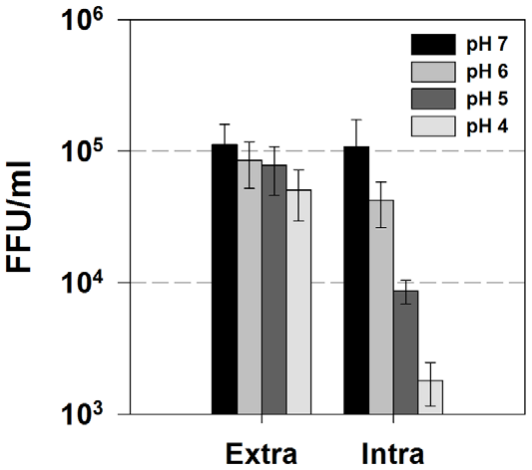
Intracellular infectious virus is acid sensitive. Huh-7.5 cells were infected with JFH-1 virus. After five days, cell culture supernatants (extracellular virus) and freeze-thaw lysates (intracellular virus) were collected and diluted 1∶1 in MES/HEPES buffer to give final pH values of 7, 6, 5 and 4. After a ten min incubation at 37°C, the viral titers were determined by FFU assay. Data are represented as mean ± SE from three independent experiments.

We have shown that p7 can dramatically alter the pH of intracellular compartments and that this vesicular alkalinization is required for infectious virus production. While it has previously been shown that mature HCV particles are acid resistant, we hypothesized that intracellular infectious virus particles might be acid sensitive and that p7-induced alkalinization could protect them from an acid milieu. To examine this, we isolated both extracellular and intracellular virus and exposed them to acidic solutions. Huh-7.5 cells were infected with cell culture passaged JFH-1 virus. After five days, both extracellular and intracellular virus was harvested and incubated for 10 min at 37°C in identical HEPES/MOPS buffer solutions to achieve final pH values of 7.0, 6.0, 5.0, and 4.0. This solution was then neutralized back to pH 7.0 and used to inoculate naïve Huh-7.5 cells. Infectious titer was determined by the standard FFU assay. Similarly to what has been reported [28], extracellular virus was acid stable and infectivity was unaffected when the virions were exposed to pH values as low as 4.0 (Fig. 10). However, intracellular infectious virions displayed greatly increased acid sensitivity, with nearly a 2 log_10_ decrease in infectivity at pH 4.0.

## Discussion

HCV p7 is required for infectious particle formation and plays a role during virion egress [16], [17]. Convincing data shows that it forms ion channels in artificial lipid bilayers [7], [8], [9], [29] yet, whether ion channel activity is required by the virus, or whether p7 functions as a channel at all inside infected cells has remained unknown. In the present study we evaluated the ability of p7 to facilitate equilibration of intracellular pH gradients and examined its contribution to the production of infectious virus. We demonstrate that p7 mediates proton (H^+^) equilibration in isolated vesicles as well as in Huh7 cells *in vivo*. Its presence results in a loss of intracellular compartment acidification, and this reduction in acidification is required for the production of infectious virus. While mature virions are acid stable, intracellular virus can be inactivated by acidic pH. In the absence of a functional p7 channel, alternative methods to alkalinize intracellular compartments can partially rescue infectious virus production.

Native vesicles without p7 expression showed a low permeability for H^+^ as evidenced by slow and incomplete pH equilibration upon exposure to a shift in extravesicular pH. When exposed to a sudden pH shift, they slowly equilibrate pH and fail to fully equilibrate over a 3 min period. In contrast, vesicles derived from cells expressing p7 from genotypes 1a, 1b or 2a HCV were capable of rapid pH equilibration, similar to normal vesicles in the presence of FCCP. This effect was inhibited in a genotype-specific manner [19] by amantadine, rimantadine and hexamethylene amiloride, and was not observed with channel-inactive p7 variants expressed to comparable levels.

To determine the extent to which p7 alters cellular ionic homeostasis, we utilized two fluorometric pH sensors that serve as pH reporters in live cells but work by different mechanisms. LysoTracker Red DND-99 is an acidotropic probe with high selectivity for acidic organelles. Although its fluorescence is largely independent of pH, it can be fixed with paraformaldehyde and serves to mark the presence of highly acidic organelles. LysoSensor Yellow/Blue DND-160 is a ratiometric pH sensor. It concentrates in organelles with less pH sensitivity, but undergoes a pH-dependent fluorescence spectrum shift that can be used to directly measure pH. Each fluorophore showed that the expression of the HCV p7 protein by replicon RNAs or during infection resulted in an alkaline shift of organelle pH and a dramatic reduction in the number of highly acidic intracellular compartments. This loss of organelle acidity only occurred in HCV infected or p7-containing cells and did not occur with electroporation of viral RNA containing the inactive channel mutant, p7KR. It was notable that loss of acidification was not restricted to one location or structure in the cell and seemed to be a global effect. To our knowledge, this is the first time a viroporin has been shown to directly induce a global loss in organelle acidity.

Our results show that the p7-associated proton conductance is inhibited by the viroporin inhibitors, amantadine and rimantadine. However, it is worthy to note that these channel blockers had very different concentration dependence in isolated vesicle systems compared with live cells. In vesicles, our results confirmed most of what has been reported for bilayers and artificial liposomes [23]. Both amantadine and rimantadine were effective at concentrations of 1 µM for genotype 1a p7 and only rimantadine was effective as a channel blocker at 1 µM for genotype 2a p7. However, in live cells we observed a different concentration dependence. We found that amantadine did not affect or restore vesicular acidity at concentrations lower than 50 µM. Rimantadine was effective at concentrations approximately 5-fold lower. The reason for the difference between isolated vesicles and cells is not clear but could be due to limited entry into cells, active cellular extrusion or intracellular binding of the compounds.

Multiple lines of evidence demonstrate that the p7-induced loss of acidification is necessary for the production of infectious virus. First, while we confirmed that the mature HCV virions are acid stable, intracellular virus is not and therefore there appears to be a phase during maturation in which protection from an acid environment is required. This idea is further supported by the observation that concentrations of the V-ATPase inhibitor bafilomycin A1, sufficient to raise the pH of acidic organelles, partially rescued infectious virus production in the null p7KR phenotype. Virus produced from the p7KR mutant virus, as a result of bafilomycin rescue, was subsequently able to enter cells by the normal viral receptor-mediated entry pathway which was inhibited by anti-CD81 antibody. This suggests that p7 may not be required for viral entry. A second line of evidence in support of a role for p7 channel activity in infectious virus production was the observation that the JFH-RR mutant virus could be rescued by expression of influenza A M2 protein. This viroporin has been shown to be a proton equilibrating channel in the secretory pathway [12], [13], but is unlikely to be able to *trans*-complement other potential HCV p7 specific functions such as viral protein-protein interactions.

A third line of evidence that supports an ion channel function for p7 in virus production is the correlation between the effects of amantadine on intravesicular pH and virus production. As shown in Fig. 7, amantadine did prevent virus production but only at the concentrations at which it was effective as an intracellular channel inhibitor in live cells. Previous studies examining the effect of amantadine on the HCV p7 protein have been difficult to reconcile. Amantadine inhibits the genotype 1 p7 channel in bilayers at low micromolar concentrations, but it does not inhibit virus production until 50–100 µM [7], [19], and it has failed to have a clinically significant antiviral effect in patients where blood concentrations of 1–2 µM are the highest that can be achieved [30]. Our results in live cells, however, clearly show that higher extracellular concentrations of amantadine are required to restore the acidic pH of intracellular vesicles in p7 expressing cells. Furthermore, this effect has the same concentration dependence as does inhibition of virus production, thus implying a direct role of p7 channel activity in virus production and supporting that amantadine would have a specific antiviral effect *in vivo* should it reach sufficient local concentration.

A notable finding in this study is that bafilomycin A1 was able to partially overcome the defect in the KR mutant but was not able to complement a p7 deletion mutant. This suggests that p7 has additional effects unrelated to its channel activity. Recent structural analysis shows that the JFH-1 p7 protein forms a hexameric structure with a luminally-facing, open orientation [31]. This exposed region is then available to provide protein-protein interaction sites. Similarly, this is seen with HIV-1 where its viroporin, Vpu, promotes HIV-1 infectivity and release of infectious virus particles through protein-protein interactions with the host such as sequestration of the virus receptor CD4 and hindering of BST-2/tetherin [32]. This supports previous data for HCV showing that incompatibilities in the sequence of NS2 in inter-genotypic HCV chimeras that limit RNA replication can be overcome by spontaneous, compensatory mutations in p7, thus providing genetic evidence that critical protein-protein interactions exist between p7 and other non-structural proteins [17], [18]. Overall, this suggests that p7 has additional functions unrelated to its channel activity. The KR mutant protein may serve these other functions and therefore can be rescued by pH manipulation, but the total absence of p7 produces more profound defects that cannot be overcome by pH changes alone. It was recently reported that the influenza M2 protein was not able to complement the absence of p7 in a novel *trans*-complementation system using a helper replicon to express the complementing protein [33]. Using a similar construct that we used in our Δp7 experiments, this study showed that a Δp7-half deletion could be complemented to varying degrees by p7, E2p7, and E2p7NS2, but not by M2. If p7 performs distinct channel-dependent and channel independent functions, then logically M2 would not be expected to restore virus production to the p7-deleted virus as it would only compensate for the loss of H^+^ channel function.

One must also consider the possibility that polyprotein processing defects, and not loss of p7 function *per se*, is the reason for failure of p7 mutants to produce virus. It has been shown that the K33A/R35A channel inactive mutant results in inefficient processing of the polyprotein within the E2-p7-NS2 region ([16] and S. Griffin, unpublished). This can then lead to the accumulation of E2-p7, p7-NS2 or E2-p7-NS2 fusion proteins or possibly inadequate levels of mature p7 protein. While it is certain that the K33A/R35A protein is channel defective, we cannot rule out the possibility that the defects in this phenotype also result from aberrant processing. However p7 is still detectable within cells infected with these viral constructs, albeit at reduced levels (S. Griffin, unpublished). This reduction in p7 levels could thus explain the low level complementation achieved with either M2 or bafilomycin A1 compared to wild-type infection, i.e. there would be less p7 available to perform its non-ion channel functions which are necessary to make virus particles.

While the step at which acidification suppresses HCV virus production remains unclear, our findings as well as others, argue that the pH sensitive step occurs after virus entry and prior to the assembly of functional virus. M2 is required for virus disassembly in the endosomes and for equilibrating the intra-luminal pH of the *trans*-Golgi network (TGN) with the cytoplasm. It has also been demonstrated that the over-expression of M2 from particular influenza strains, induces luminal dilation of the Golgi and TGN as well as delayed transport through the secretory pathway in a similar fashion as monensin [34], suggesting that M2 ion channel activity functions at the TGN to keep the pH above the threshold at which HA conformational changes occur. Like M2, p7 has been suggested to also protect nascent HCV virions from premature acid-induced conformational changes. This is supported by the fact that M2 was able to complement a channel defective HCV and rescue virus production. While p7 has not been directly connected to membrane rearrangement or organelle disruption, we cannot rule out the possibility that p7-induced membrane alterations play a role in virus production.

It has further been observed that p7 channel defective mutations have quantitatively different effects depending on the parent viral sequence and/or genotype as well as the expression system [16]. Our results in genotype HJ3-5 chimeric virus, in which the p7 sequence is derived from genotype 1a, show that the K33A/R35A mutation completely abrogates infectious virus production and can be partially restored by bafilomycin A1. However, in the context of JC1, in which the p7 sequence is derived from genotype 2a, the K33A/R35A mutation results in only a 2-log reduction in infectious virus [16]. This emphasizes the importance of strain-dependence in determining the precise quantitative consequence of p7 defects. Overall, all data agrees that p7 is vital and our new data clearly show that the channel activity is important, yet other effects such as processing defects and loss of protein-protein interactions can contribute to the specific phenotype of p7 mutations in different viral stains and culture systems.

The step at which acidification suppresses HCV virus production is not yet clear. The most widely cited hypothesis, based on analogy to influenza [12], [13] and other viruses, is that p7 protects the nascent virus from premature acid-induced conformational changes. Our finding of acid sensitivity of intracellular but not extracellular virus suggests it occurs at a step prior to final virion exocytosis. We were not able to detect infectious intracellular virus in p7KR electroporation and we observed only minimal effects of even high doses of amantadine on the ratio of intracellular to extracellular virus. These observations argue that the pH sensitive step may also occur early in the formation of intracellular infectious viral particles and not solely at the final exocytosis step.

In conclusion, we have specifically shown that p7 dramatically alters the pH equilibration in intracellular vesicles and causes a loss of acidification of multiple compartments in live cells. Intracellular virus itself is acid sensitive and virus production by a p7 mutant with defective channel function can be partially rescued by using alternative approaches to prevent intracellular acidification. This definitively demonstrates that p7 functions as an H^+^ channel in native intracellular membranes and links p7-induced pH changes to the production of infectious intracellular viral particles. While p7 channel inhibitors have yet to demonstrate clinical efficacy in the treatment of hepatitis C, our work shows that amantadine is ineffective at the concentrations achieved clinically and thus improved agents targeting the p7 channel activity could have therapeutic potential. These novel aspects and approaches to HCV will undoubtedly reveal new details of HCV-host interactions as well as therapy, as more is uncovered about the roles of this viroporin in virus assembly and release.

## Materials and Methods

### Materials

General materials were purchased from Sigma-Aldrich (St. Louis, MO) or Fisher Scientific (Pittsburgh, PA). Dulbecco's modified Eagle medium (DMEM), fetal bovine serum, L-glutamine, sodium pyruvate and geneticin were purchased from Mediatech (Manassas, VA). Penicillin-streptomycin, MEM nonessential amino acids, Opti-MEM, lipofectamine 2000, DPX (*p*-xylene-bis-pyridinium bromide), LysoSensors Yellow/Blue DND-160 and Green DND-189 and LysoTracker Red DND-99 were purchased from Invitrogen (Carlsbad, Ca). Protease inhibitor cocktail (Sigma-Aldrich, P8340) was used at 1∶100 dilution. Amantadine hydrochloride (A1260), rimantadine hydrochloride (390593), 5-(N, N-Hexamethylene) amiloride (HMA, A9561), bafilomycin A1 (B1793), monensin and nigericin were purchased from Sigma-Aldrich. Monoclonal antibody C7-50 to core protein was purchased from Affinity BioReagents and CellTiter-Blue was purchased from Promega (Madison, WI).

### Plasmids

The plasmids pcDNA-FLAGp7 and pcDNA-FLAGp7KR were described previously [15], [20]. Both contain the p7 sequence from the J4 infectious clone of HCV genotype 1b and an N-terminal FLAG tag. The pcDNA-JFH1p7 and pcDNA-JFH1p7R33A/R35A (p7RR) plasmids are similar channel active and inactive p7 expression plasmids containing the p7 sequence derived from the JFH1 clone of HCV genotype 2a. They were constructed identically to the J4 plasmids (primers available on request) introducing an N-terminal FLAG tag by QuikChange mutagenesis (Stratagene, La Jolla, CA). The parental HJ3-5 HCV plasmid, (pH-(NS2/NS3)-J/YH/QL) has been described previously [18]. The p7KR33/35AA and Δp7 (a.a. 2580-2675) mutations were introduced into the pHJ3-5 plasmid resulting in pHJ3-5(KR) and pHJ3-5(Δp7) using QuikChange mutagenesis. The pcDNA-based plasmid expressing the HPAI Hong Kong '97 156 M2 protein was kindly provided by Wendy Barclay (Imperial College, London) and described previously [35]. The region encoding M2 was excised from this plasmid and cloned into pLVX-IRES-mCherry (Clontech) to yield mCherry-M2, respectively. The channel inactive mCherry-M2(A30P) [27] was created using QuikChange mutagenesis (Stratagene, La Jolla, CA). All sequences were confirmed by DNA sequencing analysis.

### Cell Culture and Transfection

Human embryonic kidney (HEK) 293FT cells were routinely cultured in DMEM containing 10% fetal bovine serum, 100 IU/ml penicillin, 100 µg/ml streptomycin, 0.1 mM MEM non-essential amino acids, 6 mM L-glutamine, 1 mM MEM sodium pyruvate, and 500 µg/ml geneticin. Genome-length and sub-genomic genotype 1a HCV replicon cell lines were described previously [25], [36]. Full-length and sub-genomic replicon-bearing cells were cultured in DMEM supplemented with 10% fetal bovine serum, 100 IU/ml penicillin, 100 µg/ml streptomycin, 2 µg/ml blasticidin (Invitrogen) and 200 µg/ml geneticin. Full-length replicon cells were “cured” by culturing with interferon-α (200 U/ml for 4 weeks). Media used for the culture of cured replicon cell lines contained no geneticin. All transfections were carried out using Lipofectamine 2000 (Invitrogen). pcDNA-FLAGp7 was transfected into the cells according to the manufacturer's instructions. Due to expression variability, pcDNA-FLAGp7KR needed to be transfected using 1/3 the DNA concentration of wild-type pcDNA-FLAGp7 to achieve equal protein expression levels. Transfection complexes were incubated with the cells for 24 h in Opti-MEM serum-free medium (Invitrogen) before harvesting.

### RNA Transcription, Electroporation and HCV Infectivity

RNA was synthesized with T7 MEGAScript reagents (Ambion) as in Kato et al. [37] and transfected into Huh-7.5 cells by electroporation. Briefly, 10 µg of RNA was mixed with 5×10^6^ cells suspended in 250 µl of Opti-MEM media, in a cuvette with a gap width of 0.2 cm (Bio-Rad). Electroporation consisted of one pulse of current delivered by the Gene Pulser Xcell electroporation device (Bio-Rad), set at 140 V, 1000 µF, and maximum resistance. Electroporated cells were plated in complete media (DMEM supplemented with 10% fetal bovine serum, 100 IU/ml penicillin, 100 µg/ml streptomycin and 1X nonessential amino acids). Culture medium was replaced after 24 h.

For the bafilomycin A1 rescue experiments, Huh-7.5 cells were electroporated with parental HJ3-5, HJ3-5(KR), or HJ3-5(Δp7) viral RNA and equally seeded into 2 wells of a 6-well plate. At 96 h post-electroporation, the culture medium was removed and replaced with fresh, complete media containing 2.5 pM bafilomycin A1. Following an additional 24 h of incubation, supernatant medium and cell lysates were collected and assayed for infectivity. The presence of infectious HCV particles was measured by calculating focus-forming units per ml virus (FFU/ml) with various dilutions as described below. In undiluted samples, no attempt was made to remove bafilomycin from medium or lysates.

To assay viral titers, 100 µl aliquots of serial 10-fold dilutions of supernatant cell culture fluids (clarified by low-speed centrifugation) were inoculated onto naïve Huh-7.5 cells seeded 24 h previously into 8-well chamber slides (Nalge Nunc) at 2×10^4^ cells/well. Intracellular virus was isolated by freeze-thaw of cell lysates. Briefly, infected cells were washed in PBS, dislodged from the tissue culture flask with trypsin, brought to 5.0 ml with complete media and centrifuged at 400× *g* for 5 min at 4°C. The cell pellet was resuspended in 1.0 ml DMEM and subjected to 4 cycles of freeze and thaw using a methanol/dry ice bath and a 37°C water bath. Samples were then centrifuged at 10,000× *g* for 10 min at 4°C to remove cell debris. After inoculation, cells were maintained at 37°C, 5% CO_2_ and fed with 200 µl of medium 24 h later. Following 48 h additional incubation, cells were fixed in 1∶1 methanol-acetone at room temperature for 10 min, then incubated with monoclonal antibody C7-50 to core protein (Affinity BioReagents, 1∶300) for 2 h at RT, washed with PBS twice, and incubated with Alexa Fluor 488-conjugated goat anti-mouse secondary antibody (Invitrogen, 1∶500) for 1 h at RT. Infectivity was determined by calculating focus-forming units per ml of original culture medium or cell lysate (FFU/ml).

### Subcellular Fractionation and Isolation of Membrane Vesicles

293FT cells were dislodged from the tissue culture flask into ice-cold phosphate-buffered saline (PBS) and centrifuged at 1,000× *g* for 10 min at 4°C followed by two more washes in PBS. HCV replicon cells were dislodged by incubating with pre-warmed cell dissociation solution (Sigma, C5914) for 15 min followed by three washes in PBS. The cell pellet was resuspended in 1.0 ml of homogenization media (250 mM sucrose, 1 mM EDTA, 20 mM HEPES [pH 7.4], 2 mM MgCl_2_, protease inhibitors) and homogenized on ice using 25 strokes of a loose-fitting Dounce homogenizer until ∼95% of the cells were disrupted. Homogenates were cleared of nuclei and unbroken cells by centrifuging at 1,000× *g* for 10 min at 4°C. For vesicle isolation, the 1,000× *g* supernatant was centrifuged at 10,000× *g* for 15 min at 4°C to remove all heavy membranes. The resultant supernatant was centrifuged at 120,000× *g* in a MLS-50 swinging bucket rotor for 1 h at 4°C to pellet membrane vesicles. Vesicles were resuspended in homogenization buffer, flash frozen in liquid nitrogen and stored in 100 µg aliquots at −80°C until needed for conductance assays. For gradient analysis, the 1,000× *g* supernatant was centrifuged at 3,000× *g* for 10 min at 4°C to pellet heavy mitochondria and plasma membrane fragments.

### Western Blotting

Western blotting was performed using anti-Flag M2, 1 µg/ml (Sigma-Aldrich), anti-PDI, 1∶2,000 (Stressgen, Victoria, BC, Canada), anti-GRP75, 1∶20 (Affinity Bioreagents, Golden, CO), anti-LAMP2 H4B4, 1∶2,000 (DHSB, University of Iowa), anti-core, 1∶1,000 (Affinity Bioreagents) and anti-β-actin, 1∶15,000 (Sigma-Aldrich). Horseradish peroxidase-conjugated secondary antibodies were from Pierce Biotechnologies (Rockford, IL). Immunoblots were detected using the ECL Plus Western Blotting Detection System (Amersham Biosciences, Piscataway, NJ).

### Measurement of Proton Permeability

Isolated vesicles (200 µg) were thawed on ice, brought to 1.0 ml with conductance assay buffer (10 mM MOPS [pH 7.0], 150 mM KCl, 4 mM MgSO_4_) containing 5 mM HPTS (8-hydroxypyrene-1,3,6-trisulfonic acid, trisodium salt, Invitrogen) and broken and resealed with 10 strokes of a loose-fitting Dounce homogenizer to incorporate HPTS into the vesicle lumen. To separate HPTS-loaded vesicles from extra-vesicular free dye, re-homogenized vesicles were applied to a 5 ml Bio-Gel P-10 size exclusion column, with a 20 kD exclusion limit prepared as according to manufacturer's instructions (Bio-Rad). The column was eluted using HPTS-free conductance assay buffer. Fractions were collected and assayed for protein and HPTS fluorescence at ex 450 nm, em 520 nm. Protein containing fractions eluting in the void volume corresponded to a small fluorescence peak and represented HPTS-loaded vesicles. These were pooled and used for conductance assays. Protein concentration within the fractions was assayed using Bio-Rad protein assay dye reagent.

Vesicular proton permeability was measured by a modification of the method described by Grover et al. [38]. Fluorescence measurements were performed at room temperature in a Fluostar Optima plate reader equipped with an integrated syringe injector (BMG Labtech, Durham, NC). The excitation wavelengths were 450 nm (pH-dependent) and 405 nm (pH-independent) and data were collected every 1 s for 4 min at an emission wavelength of 520 nm. To determine changes of intravesicular pH, HPTS-loaded vesicles were diluted in HPTS-free conductance assay buffer, allowed to equilibrate at room temperature for 30 min, and subsequently pipetted into a 96 well plate, 100 µl per well. Fluorescence of the vesicle suspension was monitored and once a stable baseline was achieved, 10 µl of 50 mM KOH was injected per well while the plate was stirred and the fluorescence was monitored. The addition of base alkalinized the extra-vesicular environment from pH 7.0 to 8.0 as confirmed with a pH meter. Ionophore and inhibitor compounds were added to the vesicle suspension just prior to reading. Intravesicular pH was calculated from the fluorescence ratio using a bulk solution extracellular calibration curve performed with a conventional pH meter. In some experiments any extravesicular fluorescence signal was quenched by addition of 15 mM p-xylene-bis-pyridinium bromide (DPX).

### Live Cell Imaging of Vesicular pH

HCV replicon bearing, infected or electroporated cells were plated on poly-D-lysine coated cover slips and allowed to grow overnight. The cells were then washed in HEPES solution (10 mM HEPES, 133.5 mM NaCl, 2.0 mM CaCl_2_, 4.0 mM KCl, 1.2 mM MgS0_4_, 1.2 mM NaH_2_PO_4_, 11 mM glucose; pH 7.4) and loaded with 5 µM LysoSensor Yellow/Blue DND-160 diluted in HEPES solution at 37°C for 45 min. The cells were washed twice and immediately imaged. Imaging was performed using a Nikon eclipse Ti PFS Quantitative Fluorescence Live-Cell and Multidimensional Imaging System equipped with a digital monochrome Coolsnap HQ^2^ camera (Roper Scientific, Tucson, AZ). Fluorescence images were collected using Metafluor software (Universal Imaging, Downingtown, PA). Data were recorded at excitation/emission wavelengths of 340/440 nm and 380/510 nm using a 410 nm dichroic.

Cell fluorescence ratios were determined by image analysis of the stored single wavelength images using Metafluor software. For each cell a region of interest was delineated that encompassed the cytosolic space of the cell and excluded the nucleus. Ratio was calculated for each cell as R = (F_1_-B_1_)/(F_2_-B_2_) where F_1_ and F_2_ are the fluorescence intensities at 380/525 and 340/440 respectively and B_1_ and B_2_ are the corresponding background values determined from a region on the same images that was near the cell but did not contain a cell.

Calibration of the relationship between R values and pH was performed by two different methods. First, a free solution calibration was performed using LysoSensor solutions of known pH and measuring R with the microscope as described. The calibration curve was well fitted by the equation pH  =  ln[(R-Y_o_)/a] ·1/b, where Y_o_, a and b are constants. An intracellular calibration was also performed as described elsewhere [39]. Cells were loaded with LysoSensor and then permeabilized with 10 µM monensin and 10 µM nigericin. They were then serially exposed to calibration buffer solutions containing 25 mM MES, 5 mM NaCl, 115 mM KCl and 1.2 mM MgSO_4_ at fixed pH and R was measured as described. The 2 methods agreed well. The ionophores, however, resulted in loss of LysoSensor from intracellular vesicles at higher pH. This resulted in very low signals above pH 5.5 and made the intracellular calibration method impractical except for use at the most acid pH range. For this reason, the free solution calibration was used in most experiments. R values were converted to pH by extrapolation of the calibration curve.

For experiments to determine the concentration dependence of bafilomycin on vesicular pH, Huh-7.5 cells were plated on poly-D-lysine coated cover slips and allowed to grow overnight, after which time the cells were washed in HEPES solution and loaded with 2 µM of the single wavelength pH indicator LysoSensor Green DND-189 for 30 min at 37°C. The cells were washed, treated with HEPES ± increasing concentrations of bafilomycin A1 and immediately imaged. The increase in vesicular pH in response to increasing concentrations of bafilomycin A1 was determined by imaging at excitation of 443 nm and emission at 500 nM.

### Measurement of Acidic Compartments in Fixed Cells

Cells, approximately 5000, were plated on 12 mm polylysine coated glass cover slips and incubated overnight. The cells were then washed in HEPES solution and loaded with 0.5 µM LysoTracker Red DND-99 in HEPES solution for 30 min at 37°C. The cells were washed in PBS, fixed in 4% paraformaldehyde for 30 min at room temperature, permeabilized in ice-cold acetone for 5 min and incubated in IF buffer (1% BSA, 2.5 mM EDTA in PBS) for 1 hr at room temperature. HCV core protein was labeled by incubating with anti-core, 1∶300, as described above. Nuclei were counterstained by incubation for 5 min with 1.0 µg/ml DAPI and the cover slips were mounted in Fluorsave mounting medium (Invitrogen). LysoTracker positive staining was quantitated by image analysis in a Nikon TiE system as described above. Single wavelength fluorescence images were acquired at 560 nm excitation, 607 nm emission. A region of interest was chosen to include the entire area of a single cell. Images were acquired at fixed detector gain that avoided saturation in the brightest images and mean pixel intensity of each cell was determined and reported as mean cellular fluorescence intensity.

### Cytotoxicity of Bafilomycin

Huh-7.5 cells were plated in 96-well tissue culture plates at 10,000 cells per well and allowed to grow overnight after which time the cells were washed with PBS and the medium was replaced with fresh compete medium containing varying concentrations of bafilomycin A1 for 24 h. The cells were then washed in PBS and fresh DMEM containing 10% CellTiter-Blue reagent (Promega) was added. After 3 h incubation at 37°C, 5% CO_2_, the fluorescence at 560/590 nm was read in a Fluostar Optima plate reader.

### Effect of Bafilomycin A1 on Viral Entry

Huh-7.5 cells (2×10^4^ cells) were plated in the wells of an 8-well chamber slide and incubated overnight. The cells were then washed in PBS and incubated at 37°C, 5%CO_2_ in complete media containing varying concentrations of bafilomycin A1 for 1 hr. The media was then removed and the cells were infected with 0.5 ml complete media containing HJ3-5 virus (MOI 1.0) ± bafilomycin A1 and incubated at 37°C, 5% CO_2_ for 3 hr. The inoculum was then removed, the cells were washed in PBS and fresh complete media was added. Following an additional 72 hr incubation, infected cells were fixed by methanol:acetone (1∶1), stained for core protein and foci counted as described above.

### M2 Trans-complementation

Huh-7.5 cells (2.5×10^6^) were co-electroporated with 20 µg mCherry-M2 or mCherry-M2(A30P) DNA and 10 µg JFH-1 derived RNAs as described previously. The electroporated cells were resuspended into 5 ml complete media and seeded into two wells of a 6-well plate. After 48 hr, the cells were re-transfected with 20 µg mCherry-M2 or mCherry-M2(A30P) using lipofectamine according to manufacturer's instructions. 24 hr after transfection, the media was removed and fresh, complete media ±50 µM amantadine was added for an additional 24 hr. Secreted infectivity was quantified by focus forming assay by infecting 2×10^4^ naïve Huh-7.5 cells seeded the preceding day in an 8-well chamber slide. Infected cells were fixed by methanol:acetone (1∶1), stained for core protein and foci counted as described above.

### Low pH Treatment of Extracellular and Intracellular Virus

Huh-7.5 cells were plated in a T-175 cm^2^ culture dish and infected with cell culture passaged JFH-1 virus at an MOI of 1.0. After five days, the cell supernatant fluids were collected and clarified by low-speed centrifugation. Intracellular virus was obtained by freeze-thaw of washed cellular pellets as described above. Extracellular and intracellular viral stocks were then identically diluted 1∶1 in a HEPES/MES buffer (20 mM HEPES, 20 mM MES, 133.5 mM NaCl, 2.0 mM CaCl_2_, 4.0 mM KCl, 1.2 mM MgS0_4_, 11 mM glucose; pH 7.4) at pH 2.1, 2.6, 5.3, or 7.0. The final pH values of the virus/buffer solutions were 4.0, 5.0, 6.0, and 7.0 respectively. The virus/buffer suspensions were incubated in a 37°C water bath for 10 min and then neutralized with 1M NaOH. The samples were then clarified by low-speed centrifugation at 500× *g* for 5 min and diluted 1∶10 or 1∶100 in complete media. Infectivity was determined by calculating focus-forming units per ml of original culture medium or cell lysate (FFU/ml).

### Statistics

Results are expressed as mean ± SE. The Student *t* test was used for statistical analyses. P≤0.05 was considered significant.
